# FreeContact: fast and free software for protein contact prediction from residue co-evolution

**DOI:** 10.1186/1471-2105-15-85

**Published:** 2014-03-26

**Authors:** László Kaján, Thomas A Hopf, Matúš Kalaš, Debora S Marks, Burkhard Rost

**Affiliations:** 1 Department for Bioinformatics and Computational Biology, TU Munich, Boltzmannstraße 3, Garching 85748, Germany; 2 Department of Systems Biology, Harvard Medical School, Boston, Massachusetts, USA; 3 Computational Biology Unit, Uni Computing, Bergen 5008, Norway; 4 Department of Informatics, University of Bergen, Bergen 5008, Norway; 5 Institute of Advanced Study (TUM-IAS), Lichtenbergstr. 2a, Garching/Munich 85748, Germany; 6 WZW – Weihenstephan, Alte Akademie 8, Freising, Germany

**Keywords:** Protein structure prediction, Protein sequence analysis, Fast protein contact prediction, 2D prediction, Open-source software, EVfold, EVcouplings, PSICOV, mfDCA, BioXSD, Debian package

## Abstract

**Background:**

20 years of improved technology and growing sequences now renders residue-residue contact constraints in large protein families through correlated mutations accurate enough to drive *de novo* predictions of protein three-dimensional structure. The method EVfold broke new ground using mean-field Direct Coupling Analysis (EVfold-mfDCA); the method PSICOV applied a related concept by estimating a sparse inverse covariance matrix. Both methods (EVfold-mfDCA and PSICOV) are publicly available, but both require too much CPU time for interactive applications. On top, EVfold-mfDCA depends on proprietary software.

**Results:**

Here, we present *FreeContact*, a fast, open source implementation of EVfold-mfDCA and PSICOV. On a test set of 140 proteins, *FreeContact* was almost eight times faster than PSICOV without decreasing prediction performance. The EVfold-mfDCA implementation of *FreeContact* was over 220 times faster than PSICOV with negligible performance decrease. EVfold-mfDCA was unavailable for testing due to its dependency on proprietary software. *FreeContact* is implemented as the free C++ library “libfreecontact”, complete with command line tool “freecontact”, as well as Perl and Python modules. All components are available as Debian packages. *FreeContact* supports the BioXSD format for interoperability.

**Conclusions:**

*FreeContact* provides the opportunity to compute reliable contact predictions in any environment (desktop or cloud).

## Background

Experimental high-resolution three-dimensional (3D) structures are available for fewer than one percent of all known proteins of known sequence (March, 2014: 100 k protein structures in the PDB [1] vs. 52 M sequences in UniProt [2]), and this sequence-structure gap [3] continues to increase. Homology modeling [4] or comparative modeling [5] is the only bridge that allows reliable modeling of 3D structure for about 20-40% of the residues in all known proteins [6]. This boost of experimental information constitutes an immense achievement of computational biology (with about $50 billion dollars investment for experimentally unraveling 0.1 M known structures, computational biology generates structural knowledge for another ~20 M for just a few million).

Over the last two years, methods have been introduced that for the first time enable reliable *de novo* prediction of 3D structure for large proteins, i.e. intrudes into realms unreachable by comparative modeling [7]. EVfold [8,9] has been succeeding in sustained and reliable predictions of two-dimensional (2D) inter-residue contacts, i.e. the prediction of which residue pairs are near each other in the native protein structure. The success and elegance of the contact prediction through mean-field direct-coupling analysis [10] of EVfold (EVfold-mfDCA) has revived the field (e.g. PSICOV [11], plmDCA [12], PconsC [13] and PhyCMAP [14-16], and EVfold_membrane [7]). Wider application of this new generation of contact prediction tools is enticing, but currently hampered by two problems.

The first problem relates to the amount of sequence information needed, e.g. EVfold tends to perform better with 50 k sequences in a family than with 10 k. To put this into perspective: a decade ago, only the 10% largest families had over 100 homologues [17], now we have a method for which 100 times this may no longer suffice. This is why, e.g. EVfold_membrane can predict structures for only tens of families [7]. However, these families are so gigantic that they cover some 3-6% of all known sequences.

The second problem pertains to the speed of the new methods and their ease of availability. EVfold-mfDCA is publicly available, but its implementation requires proprietary interpreter software. PSICOV has recently been released under the GPLv2 (version 1.09 and later), but is not optimized for speed (as of version 1.10). Runtimes often exceed tens of minutes (using the optimal parameters published). This might be restrictive for large-scale data analysis and for public web service operations such as PredictProtein [18]. Neither EVfold-mfDCA nor PSICOV are packaged for convenient installation and usage.

Here, we report the release of *FreeContact*, a freely available software that considerably reduces the runtime for EVfold-mfDCA and PSICOV and provides convenient Debian [19] packages freely, open-source available to all users.

## Implementation

FreeContact is an open-source EVfold-mfDCA implementation optimized for speed. FreeContact can also be parameterized to produce results according to the PSICOV algorithm, because these two methods share many computational steps. For optimization, we identified the following program components that contribute significantly to runtime: BLOSUM-style weighting [20] of protein sequences in the input alignment (shared by EVfold-mfDCA and PSICOV), counting pairwise residue frequencies (also shared), shrinking the covariance matrix (PSICOV), sparse inverse covariance estimation (PSICOV), and covariance matrix inversion (EVfold-mfDCA).

### Speed-up

Sequence weights result from computing the percentage sequence identity between each protein pair in a family. Our implementation uses standard ×86-64 architecture streaming SIMD instructions (single instruction, multiple data) extensions 2 (SSE2). These instructions operate on 128-bit registers, allowing the simultaneous comparison of 16 residues (each represented in a byte). A generic implementation is provided for architectures without SSE2 instructions. Both implementations benefit from multiple cores using OpenMP [21] to parallelize loops. Parallelization of loops through OpenMP also accelerated the computation of pairwise amino acid frequencies. The usage of single-precision LAPACK [22] routines accelerated the shrinking of the covariance matrix. We used GLASSOFAST [23], a new, fast implementation of the GLASSO algorithm [24] for sparse inverse covariance matrix estimation, developed in part for its usefulness in protein contact prediction. GLASSOFAST was used with single-precision numbers and GNU Compiler Collection (GCC) auto-vectorization (“-ftree-vectorize”, implied by “–O3”). LAPACK routines (single precision) inverted the covariance matrix.

### Parameters

FreeContact is controlled by the following parameters (Table 1). Command-line usage of FreeContact is facilitated by Bash auto-completion [25] for its parameters and their arguments.

**Table 1:** FreeContact command-line parameters

--clustpc	BLOSUM-style sequence clustering percentage [0–100]
--cov20	when true, one amino acid is left out when forming the covariance matrix, making it non-overdetermined [10] [Boolean]
--density	target precision matrix density [0–1]
--estimate-ivcov	perform inverse covariance matrix estimation instead of matrix inversion [Boolean]
--apply-gapth	exclude alignment columns with a weighted gap frequency greater than --gapth from the covariance matrix [Boolean]
--gapth	weighted gap frequency threshold (0–1]
--icme-timeout	inverse covariance matrix estimation timeout in seconds [0-)
--mincontsep	minimum sequence separation (j - i ≥ arg) for reporting contacts [1-)
--pseudocnt	pseudo-count for sequence weighting [0-)
--pscount-weight	pseudo-count weight for sequence weighting [0–1]
--rho	initial value of GLASSO regularization parameter [0-)
--parprof	parameter profile selection [evfold|psicov|psicov-sd]

FreeContact command-line parameters controlling contact prediction.

We provide the choice of parameter profiles through the “--parprof” command-line option to conveniently set FreeContact options to recommended parameterizations of EVfold-mfDCA and PSICOV: the “evfold” argument sets EVfold-mfDCA compatibility mode, while “psicov” sets PSICOV “improved results” compatibility mode, and “psicov-sd” sets PSICOV “sensible default” compatibility mode (as defined in the README file distributed with PSICOV). This allows FreeContact to function as an accelerated replacement for both EVfold-mfDCA and PSICOV.

### Differences

One of the differences between the FreeContact and the original implementation of PSICOV is the interpretation of the BLOSUM-style clustering percentage for sequence weighting. The original implementation groups sequences with a similarity larger than (>) the given threshold, while FreeContact groups at larger-or-equal (≥) the threshold. This technical detail matters as it allows FreeContact to share this program component between its implementations of PSICOV and EVfold-mfDCA. The performance of PSICOV is affected minimally by this detail [see Additional file 1].

A novel addition is the optional time limit that FreeContact can impose on inverse covariance estimation. The rationale was the observation that this estimation can take exceedingly long. If the time limit is exceeded, the prediction aborts with a dedicated error code.

An important difference between the original EVfold-mfDCA and its FreeContact implementation is the way in which contact scores are computed. FreeContact returns improved “corrected norm” scores [12] instead of the original “direct information”. The reason for this is that “corrected norm” scores have been reported to be superior [12].

### Build

FreeContact was compiled with the GNU Compiler Collection (GCC, version 4.7.2, with the “–O3 -ffast-math -funroll-loops” flag), and it was linked with the threaded version of the linear algebra software ATLAS [26] (version 3.8.4, built on the host architecture). ATLAS provides a highly efficient machine-specific implementation of BLAS [27] and LAPACK [22]; it automatically adapts itself during the build process to the host architecture in order to optimize performance. FreeContact can be linked with other BLAS and LAPACK implementations. PSICOV was compiled with the recommended “-m64 –O3 -mfpmath = sse -msse3 -funroll-loops -ffast-math” options.

### Availability

FreeContact is available under the GNU General Public License version 3 or later (GPLv3+, granting freedom to use the software, guaranteeing included source code, allowing modifications, and allowing free redistribution [28]). It is available as a C++ library (called “libfreecontact”), along with a command-line executable (called “freecontact”), and modules in Perl (“FreeContact”, packaged as “libfreecontact-perl”) and Python (“freecontact”, packaged as “python-freecontact”). The library, executable, modules, and documentation are available as official Debian packages for Debian and derivative operating systems [29] - such as Ubuntu, Bio-Linux [30] and CloudBioLinux [31] - from Debian Med [32,33]. All packages can be easily installed with the common package management tools. TAR GZ downloads are available from the Rostlab FTP site [34].

## Results and discussion

PSICOV has two notable run modes: “improved results” and “sensible default”. The first (*improved results*) has been reported to be slightly more accurate and 2–3 times slower than the second [11]. We have tested the runtime of the FreeContact implementation (FC) of these modes (FC.psicov and FC.psicov-fast), and of EVfold-mfDCA (FC.evfold) on 140 proteins of the published test set of PSICOV [see Additional files 2 and 3]. We compared runtimes to PSICOV version 1.10. The original implementation of EVfold-mfDCA was unavailable for testing due to its dependency on proprietary software. Ten proteins of the complete PSICOV test set of 150 were excluded from the evaluation, because at least one of the methods failed to return results, due to either excessive run time, or insufficient total alignment weight. PSICOV was allowed to run for at least three hours. FreeContact was run with the default 30-minute time limit on the inverse covariance estimation step. All tests of FreeContact were carried out using the “FreeContact” Perl module, on a computer with 32 GB RAM and two 6-core AMD Opteron 2431 processors running at 2.4 GHz. FreeContact was run on a single thread unless indicated otherwise.

### Performance

**Figure 1:**
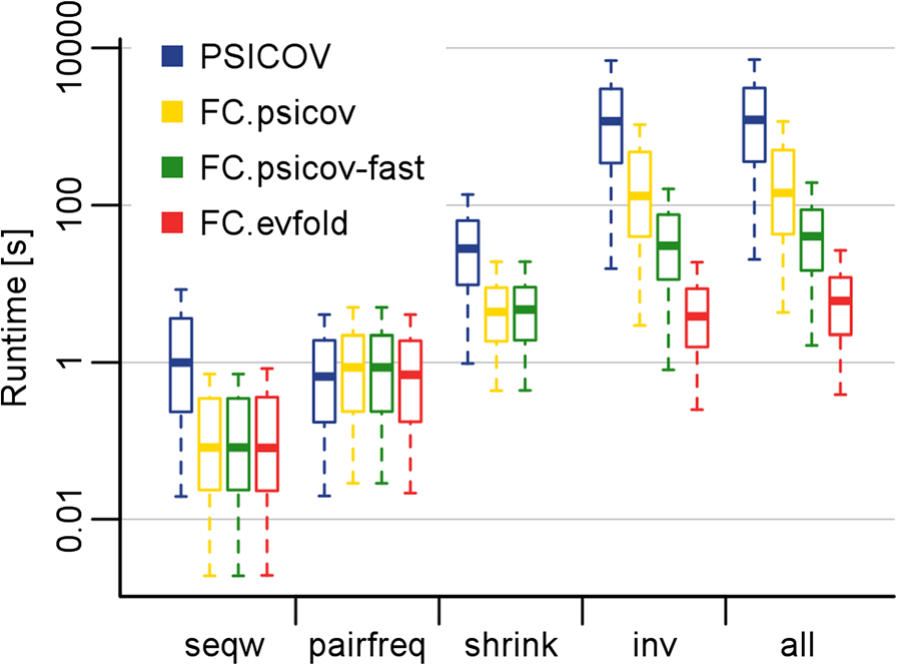
**Runtimes for FreeContact.** We measured the runtime (logarithmic y-axis) for different program components (x-axis) on a single thread. The program components were: “seqw” – sequence weighting; “pairfreq” – pairwise residue frequencies; “shrink” – shrinking of covariance matrix; “inv” – sparse inverse covariance estimation/covariance matrix inversion. The different colors distinguish: the original PSICOV implementation (blue), our acceleration of PSICOV (FC.psicov, yellow), our acceleration of the faster PSICOV version “sensible default” (FC.psicov-fast, green), and our implementation of EVfold-mfDCA (FC.evfold, red). The whiskers on the box plots show the most extreme data point that is less than 1.5-times the interquartile range from the box. Outliers are not shown. Total runtime of all methods tested is dominated by the sparse inverse covariance estimation/covariance matrix inversion component.

The most time-consuming step of the original PSICOV implementation is the sparse inverse covariance matrix estimation. In fact, this step is responsible for a large fraction of the runtime. The next most CPU-intensive steps are shrinking the covariance matrix, sequence weighting, and pairwise residue frequency calculation (Figure 1).

**Figure 2:**
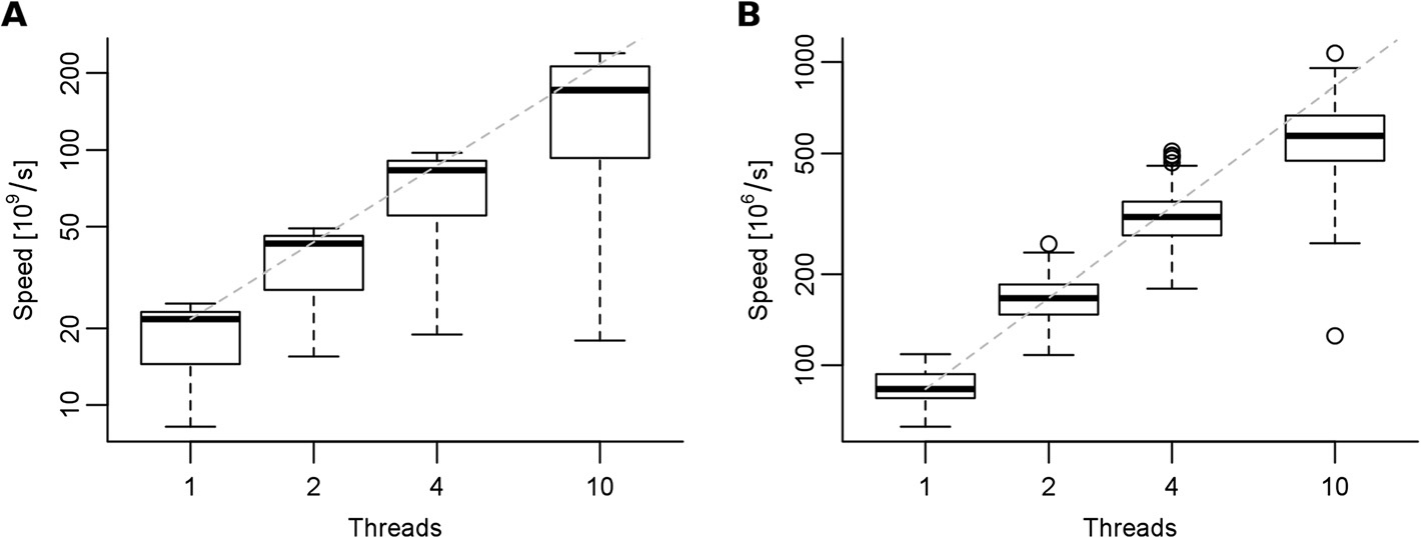
**Speedup using multiple threads. A**: Sequence weighting. Speed is calculated as: proteins in alignment^2^ length of target protein/runtime. **B**: Pairwise residue frequency calculation. Speed is calculated as: proteins in alignment length of target protein^2^/runtime. Dashed lines indicate linear correlation, extrapolated from one thread. The whiskers extend to the most extreme data point that is less than 1.5-times the interquartile range from the box. The surprisingly clear correlation between the number of threads and speed demonstrates how well our implementation scales for multi-threading.

Sequence weighting in FreeContact was accelerated over 12-fold on a single thread, compared to PSICOV (Figure 1, “seqw”). Parallelization yielded further speedup. On the 12-core test machine, 2.0, 3.8, and 7.9-fold average speedups were observed when using FreeContact with 2, 4, and 10 threads, respectively (Figure 2A). The FreeContact computation of pairwise residue frequencies was as fast as PSICOV on a single thread (Figure 1, “pairfreq”). Parallelization yielded 2.0, 3.7, and 6.8-fold speedup on 2, 4, and 10 threads, respectively (Figure 2B).

Our code, taking advantage of the optimized ATLAS implementation of single precision LAPACK routines, sped up the covariance matrix shrinking step over five-fold, compared to PSICOV (Figure 1, “shrink”). FC.psicov performed the inverse covariance matrix estimation step on average 8-times faster than PSICOV (Figure 1, “inv”), due to the optimized GLASSOFAST routine. Overall, FC.psicov, FC.psicov-fast, and FC.evfold were 7.9, 32, and 226-times faster than PSICOV on a single thread, respectively (Figure 1, “all”).

### Precision

We measured the performance of FreeContact in the following way. Two residues were defined to be in contact when their Cβ-Cβ distance (Cα-Cα for glycine) was below 8 Å (0.8 nm). In this debatable threshold, we followed the procedure introduced by the Critical Assessment of protein Structure Prediction (CASP) [35]. It was also used for the original PSICOV publication [11]. Similarly, we monitored a score that had also been introduced by CASP, namely the precision in contact predictions for pre-defined thresholds in the number of contacts predicted. The thresholds were chosen as the top L/n (L: length of target protein) contacts, with n = (1, 2, 5, 10). We distinguished different regions of sequence separation (residues j and i separated by at least [j - i] > sep, with sep = (4, 8, 11, 23)). All of those choices followed the CASP procedure. Many readers might argue for problems with those choices. However, for our purpose the CASP-like evaluation of contact prediction sufficed to establish that the FreeContact implementation of PSICOV and EVfold-mfDCA did not come at the cost of performance.

Our assessment showed that the re-implementation of PSICOV and the switching of some calculations to single precision did not significantly affect precision (Table 2: PSICOV vs. FC.psicov) [see Additional file 1]. The small differences observed were entirely caused by switching the relational operator in sequence weighting from larger (“>”) in the original PSICOV to larger-equal (“≥”) in FC.psicov (our implementation). Switching back to the original larger (“>”) resulted in identical precision [see Additional files 4 and 5].

**Table 2:** Mean precision values [%]

	**[j - i] > 4**				**[j - i] > 8**			
	**L**	**L/2**	**L/5**	**L/10**	**L**	**L/2**	**L/5**	**L/10**
PSICOV	46	60	73	78	42	58	71	77
FC.psicov	46	60	73	77	42	57	71	77
FC.psicov-fast	44	58	72	77	41	55	70	76
FC.evfold	45	57	67	73	*44*	57	69	75
	[j - i] > 11				[j - i] > 23			
	L	L/2	L/5	L/10	L	L/2	L/5	L/10
PSICOV	40	55	70	77	33	47	65	73
FC.psicov	40	55	70	76	33	47	65	73
FC.psicov-fast	39	53	68	76	32	45	63	71
FC.evfold	*42*	*56*	69	75	*35*	*49*	64	72

Mean precision values [%] for the top-L/n, L = length of target protein, n = (1, 2, 5, 10) contacts divided by sequence separation ranges [j - i] > sep, j, i residue positions, sep = (4, 8, 11, 23), where the Cβ-Cβ distance (Cα-Cα for glycine) is less than 8 Å.

We could not compare the performance of the original implementation of EVfold-mfDCA with ours (FC.evfold), due to the former’s dependency on proprietary software. We noted, however, that FC.evfold was the fastest of the four methods tested, and assessed on the PSICOV test set, the immense gain in speed appeared to come with good performance: FC.evfold outperformed the other three methods at certain top-L contact and sequence separation ranges (Table 2, *italic* values).

### Interoperability

FreeContact is not limited to the command line or C/C++ programs. Its full speed and features are available to Perl and Python scripts as well, through extension modules distributed with the software. FreeContact supports BioXSD [36] – the proposed XML data-exchange format for sequences, alignments, and features – as an option for output formats. This facilitates its integration into workflows and incorporation into Web services. We plan to support BioXSD input as well, and release a FreeContact Web service in the near future.

## Conclusions

FreeContact is a fast replacement for EVfold-mfDCA and PSICOV, offering significant acceleration on common hardware. The implementation takes full advantage of standard ×86-64 features such as SSE2 instructions and multiple cores. The speed increase is important for large-scale protein contact or protein-protein interaction prediction projects, as it leads to significant savings through shorter runtimes. We anticipate providing updates to FreeContact as the algorithms are developed.

## Availability and requirements

• Project name: FreeContact

• Project home page: http://rostlab.org/owiki/index.php/FreeContact

• Operating systems: UNIX-like (tested on Debian and Ubuntu)

• Programming language: C++, Fortran

• Other requirements: Autotools, Boost C++ Libraries, BLAS, LAPACK, Xerces C++, CodeSynthesis XSD (the latter two are required only for optional XML I/O)

• License: GPLv3 or later

• Any restrictions to use by non-academics: none

## Abbreviations

2D: Two-dimensional; 3D: Three-dimensional; BMBF: Bundesministerium fuer Bildung und Forschung; CASP: Critical assessment of protein structure prediction; EBI: European bioinformatics institute; FC: FreeContact; GCC: GNU compiler collection; GPLv3+: GNU general public license version 3 or later; PDB: Protein data bank; SIB: Swiss institute of bioinformatics; SIMD: Single instruction multiple data; SSE2: Streaming SIMD extensions 2; TUM: Technical university of Munich, Germany.

## Competing interests

The authors declare that they have no competing interests.

## Authors’ contributions

LK programmed FreeContact, created extension modules, packaged the software, coordinated the project and drafted the manuscript; TAH advised on EVfold-mfDCA implementation and reviewed the manuscript; MK contributed to the development of the FreeContact XML schema, advised on interoperability, reviewed and edited the manuscript; DSM reviewed and edited the manuscript; BR contributed to the concept, reviewed and edited the manuscript. All authors read and approved the final manuscript.

## Authors’ information

LK is a Debian Developer and an active member of the Debian Med [33] team.

## Supplementary Material

Additional file 1 **FC.psicov vs. PSICOV precision plot.** Precision of FC.psicov plotted against PSICOV, for the test set of 140 proteins. Precision values for the top-L, L = length of target protein, contacts with separation range [j - i] > 4, where the Cβ-Cβ distance (Cα-Cα for glycine) is less than 8Å. Click here for file

Additional file 2 **PDB codes of target proteins.** List of 140 PDB codes of target proteins used for testing. A subset of the test protein set of PSICOV. Click here for file

Additional file 3 **Distribution of target protein alignment sizes and lengths.** Alignment size of the 140 target proteins plotted against the target sequence length. Click here for file

Additional file 4 **FC.psicov> vs. PSICOV precision plot.** Precision of FC.psicov> plotted against PSICOV, for the test set of 140 proteins. FC.psicov> uses “>“ for the sequence clustering threshold, like PSICOV. Precision values for the top-L, L = length of target protein, contacts with separation range [j - i] > 4, where the Cβ-Cβ distance (Cα-Cα for glycine) is less than 8Å. Click here for file

Additional file 5 **FC.psicov+ vs. PSICOV precision plot.** Precision of FC.psicov+ plotted against PSICOV, for the test set of 140 proteins. FC.psicov+ is FC.psicov (using “≥”), run with slightly higher sequence clustering thresholds to compensate for the “>“ comparison used by PSICOV. Precision values for the top-L, L = length of target protein, contacts with separation range [j - i] > 4, where the Cβ-Cβ distance (Cα-Cα for glycine) is less than 8Å. Click here for file
