# Supplementary figures and images for: FreeContact: fast and free software for protein contact prediction from residue co-evolution
Source: BMC Bioinformatics. 2014 Mar 26;15:85. doi: 10.1186/1471-2105-15-85 (PMC3987048; doi:10.1186/1471-2105-15-85)

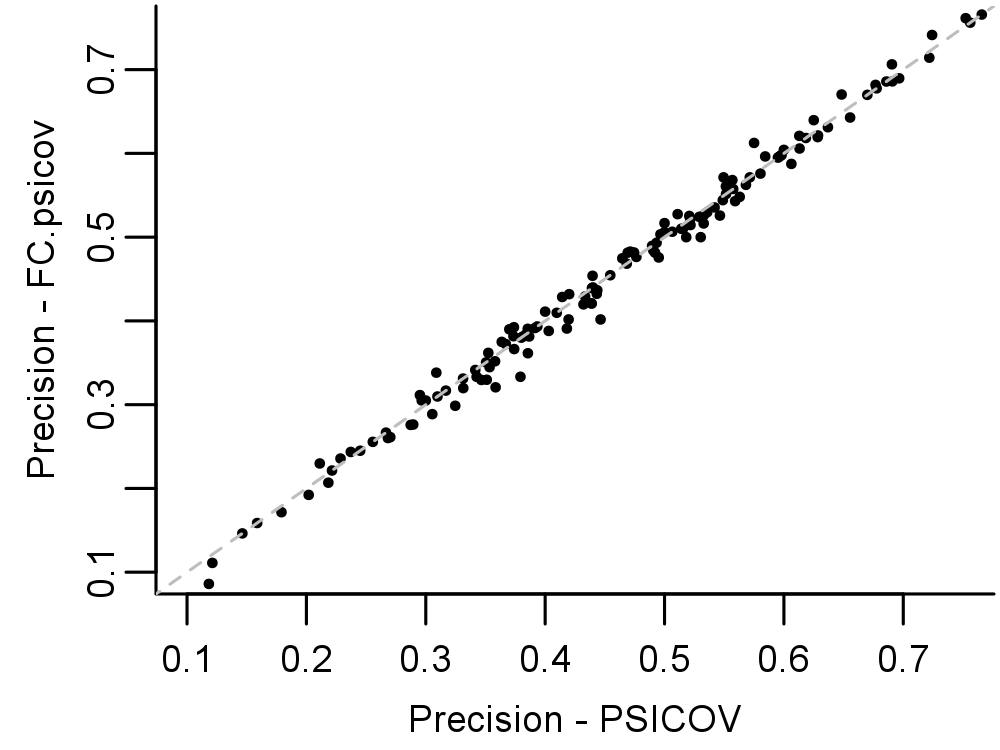

Supplement: Additional file 1 — FC.psicov vs. PSICOV precision plot. Precision of FC.psicov plotted against PSICOV, for the test set of 140 proteins. Precision values for the top-L, L = length of target protein, contacts with separation range [j - i] > 4, where the Cβ-Cβ distance (Cα-Cα for glycine) is less than 8Å. [file 1471-2105-15-85-S1.png]

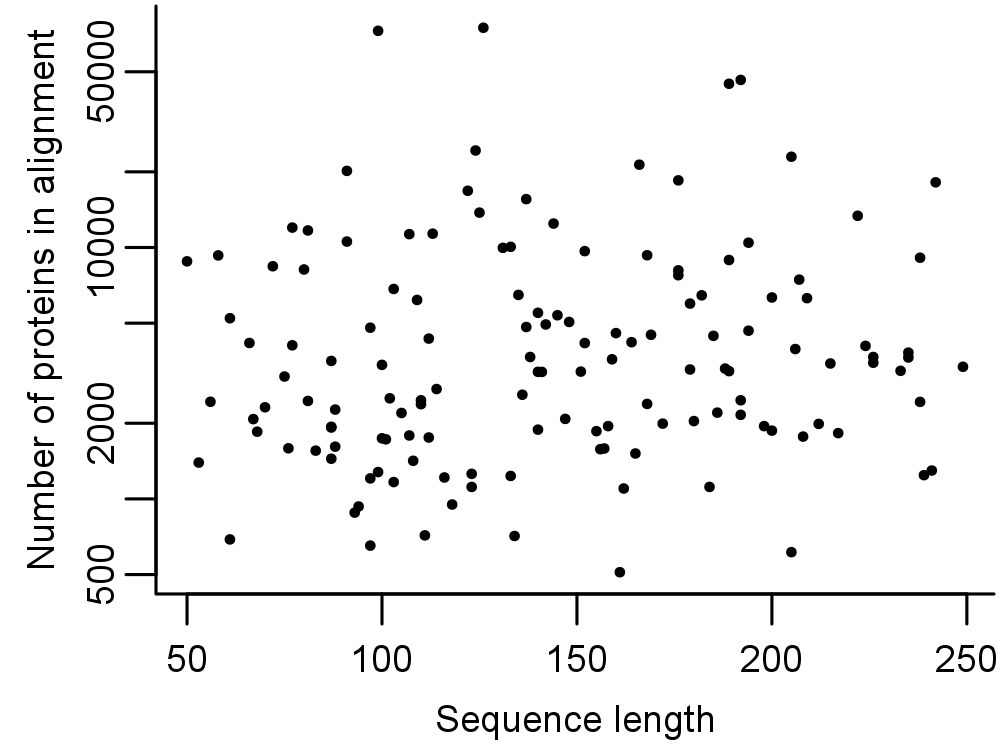

Supplement: Additional file 3 — Distribution of target protein alignment sizes and lengths. Alignment size of the 140 target proteins plotted against the target sequence length. [file 1471-2105-15-85-S3.png]

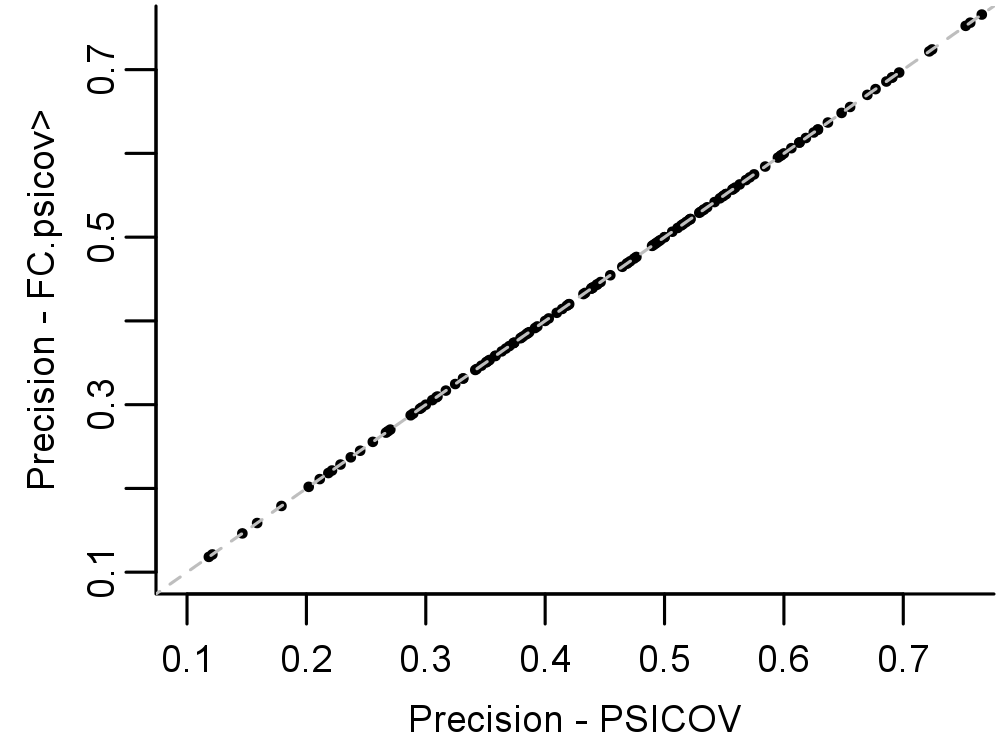

Supplement: Additional file 4 — FC.psicov> vs. PSICOV precision plot. Precision of FC.psicov> plotted against PSICOV, for the test set of 140 proteins. FC.psicov> uses “>“ for the sequence clustering threshold, like PSICOV. Precision values for the top-L, L = length of target protein, contacts with separation range [j - i] > 4, where the Cβ-Cβ distance (Cα-Cα for glycine) is less than 8Å. [file 1471-2105-15-85-S4.png]

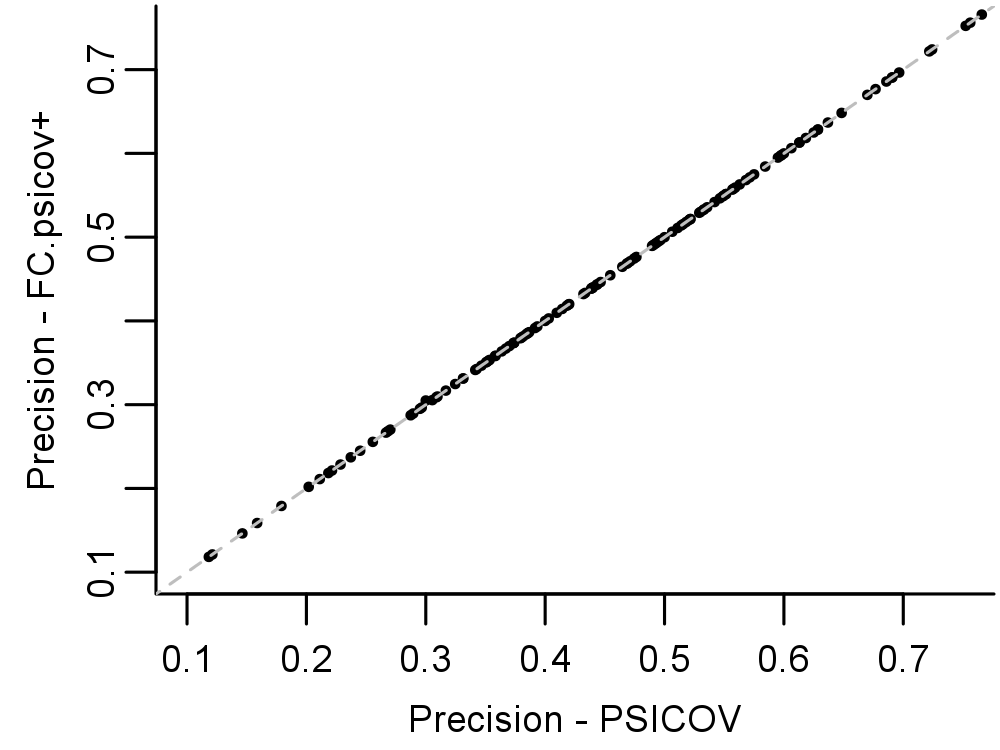

Supplement: Additional file 5 — FC.psicov+ vs. PSICOV precision plot. Precision of FC.psicov+ plotted against PSICOV, for the test set of 140 proteins. FC.psicov+ is FC.psicov (using “≥”), run with slightly higher sequence clustering thresholds to compensate for the “>“ comparison used by PSICOV. Precision values for the top-L, L = length of target protein, contacts with separation range [j - i] > 4, where the Cβ-Cβ distance (Cα-Cα for glycine) is less than 8Å. [file 1471-2105-15-85-S5.png]
